# Thoracic Ultrasound for Acute Dyspnea in Interstitial Lung Disease

**DOI:** 10.3390/jcm14124159

**Published:** 2025-06-11

**Authors:** Betsega A. Bayeh, Sylvain Marchand-Adam, Sylvie Legué, David Luque Paz, Laurent Plantier, Thomas Flament

**Affiliations:** 1 Tours University Hospital, Centre de Référence des Maladies Pulmonaires Rares de la Région Centre-Val-de-Loire, Hôpital Bretonneau, 37000 Tours, France; s.marchandadam@univ-tours.fr (S.M.-A.); s.legue@chu-tours.fr (S.L.); l.plantier@chu-tours.fr (L.P.); t.flament@chu-tours.fr (T.F.); 2 Faculté de Médecine, Tours University, Centre d’Étude des Pathologies Respiratoires (CEPR) INSERM U1100, 37032 Tours, France; 3 Lung Ultrasound Working Group (G-ECHO), Société de Pneumologie de Langue Française, 75935 Paris, France; 4 Infectious Diseases and Intensive Care Unit, Rennes University Hospital, 35000 Rennes, France; david.luquepaz@live.fr

**Keywords:** lung ultrasound, interstitial lung disease, dyspnea, exacerbation, infection

## Abstract

**Background:** Lung ultrasound (LUS) can be used at follow-up for patients with stable interstitial lung disease (ILD). LUS could also help guide the diagnosis of etiology for acute respiratory episodes. **Methods:** We conducted a prospective, one-center, observational study including patients with ILD hospitalized in the pulmonology unit or in the intensive care unit of the Tours University Hospital for acute dyspnea. LUS was performed at admission and then at a follow-up visit in the six months following discharge. We compared the number of B-lines between the two LUSs. We also compared the features of the first LUS between the different etiologies responsible for increased dyspnea. **Results:** Of 24 patients, 16 had acute ILD exacerbation (67%), 6 had pulmonary infections (25%) and 2 had acute heart failure (8%). LUS was feasible in all patients and always showed lung sliding, pleural irregularities and B-lines. There were pleural effusions in four cases (17%) and pulmonary consolidations in two cases (8%). Seven patients had A-lines in at least one thoracic space on the initial LUS. We found a significant decrease in the number of B-lines at follow-up (76; IQR, [59–86.75]) compared to admission (86.5; IQR, [71.5–94.5]) (*p*-value = 0.02). There was a trend of more A-lines in patients with infection (1 [0.25–1.75]) compared to AE-ILD (0 [0–0]). **Conclusions:** Following an episode of acute dyspnea in patients with ILD, LUS shows a decrease in the number of B-lines. Patients with ILD and concurrent pulmonary infection may have more A-lines than patients with AE-ILD.

## 1. Introduction

Interstitial lung disease (ILD) refers to a heterogeneous group of diseases with varying causes and whose commonality is a disorganization of the pulmonary architecture. ILD may be due to inflammatory causes such as genetic mutations, connective tissue disorders (CTDs) and external systemic or inhaled factors or may be idiopathic [1]. The natural evolution of most patients with ILD is a gradual decline in lung function resulting in an increase in dyspnea. Acute worsening of dyspnea is relatively common and can be attributed to many etiologies, with acute exacerbation of the underlying ILD, infection, acute heart failure (AHF), pulmonary embolism (PE) and pneumothorax being the most frequent [2]. French practical guidelines recommend performing imaging such as chest computed tomography (chest CT) when patients are hospitalized for increased dyspnea. This allows a thorough evaluation of the lung parenchyma and exploration of the pulmonary vasculature in order to rule out PE [3]. Though chest CT is very informative, it exposes the patient to radiation and the nephrotoxicity of contrast agents. Several criteria are required to obtain good-quality imaging: the patient must be in apnea and forced inspiration, tolerate transport and in a supine position.

Lung ultrasound (LUS) is used in emergency settings in case of respiratory failure. Its use is widespread for the diagnosis of pleural effusion, pneumothorax [4], infectious pneumonia [5] and AHF [6]. Lichtenstein et al. developed an algorithm known as the BLUE protocol to guide the use of LUS in intensive care unit (ICU) patients with acute respiratory failure [4]. Bekgoz et al. showed that the BLUE protocol could be used in similar patients in the emergency department [5]. To this day, studies evaluating the relevance of LUS in acute dyspnea have excluded patients with pre-existing ILD as these diseases are responsible for the increase in B-lines, which could be a confounding factor.

The use of LUS in ILD has been the subject of multiple publications in patients with idiopathic pulmonary fibrosis (IPF) [6], systemic sclerosis [7], rheumatoid arthritis [8] and other connective tissue diseases [9]. LUS signs in patients with ILD include the following: B-lines and irregularity and/or thickening of the pleural line [10]. LUS is convenient as it can be performed rapidly, at the patient’s bedside, in a supine or sitting position, without radiation and without apnea. In addition, physicians can be easily trained to perform LUS at the bedside. In fact, pulmonologists in most French university hospitals are trained to perform LUSs during residency.

The main objective of our single-center pilot study was to describe the characteristics of LUS in patients with ILD during hospitalization for acute dyspnea and after the stabilization of their respiratory status. The secondary objective was to compare LUS features between the main identified etiologies of acute dyspnea.

## 2. Materials and Methods

The Thoracic Ultrasound for Acute Dyspnea in Interstitial Lung Disease (TURTLE) is a prospective, observational, monocentric and pilot study.

### 2.1. Patient Recruitment

We included all patients (age > 18 years) with pre-existing ILD (*n* = 21) or the discovery of ILD during their hospital stay (*n* = 3), hospitalized in the respiratory unit or in the ICU of Tours University Hospital from 1 November 2022 to 30 April 2023 for an increase in dyspnea in the 4 weeks preceding hospital admission.

Non-inclusion criteria were as follows: (i) post-radiotherapy ILD, drug-induced ILD or pulmonary fibrosis secondary to sarcoidosis; (ii) thoracic malignancy; (iii) pregnancy or breastfeeding; (iv) chest tube in place for pneumothorax at the time of inclusion (i.e., the first LUS).

In order to not distort the results of the LUS, we excluded patients who had received diuretics over 6 h before the first LUS if the final diagnosis was AHF [11] as well as patients who had received antibiotics over 48 h before the first LUS if the final diagnosis was infectious pneumonia.

### 2.2. Ultrasound Protocol

LUS was performed using a Sparq ultrasound system (Philips Healthcare, Best, The Netherlands, in operation since 26 October 2018) with a convex probe (1–5 MHz) by one of two certified operators, blind to all other exam results. In the absence of a lung sliding sign, a complimentary LUS using a high-frequency (10–15 MHz) linear probe was performed.

We explored 14 thoracic areas: 2 anterior, 2 axillary and 3 posterior on each side [12]. The patient was placed in a supine or sitting position. The presence of lung sliding, pleural irregularity, pleural effusion or pulmonary consolidation and the quantification of A-lines and B-lines were recorded in each thoracic area. Each thoracic area was recorded for 6 s. All LUS images were reviewed by a blinded certified reader.

The first LUS (LUS1) was performed upon admission.

A second LUS (LUS2) was performed during a follow-up appointment with the patient’s pulmonologist in the six months following their hospital stay. LUS2 was performed in the same conditions as the first one, and the same information was collected.

### 2.3. Data Collection

Medical files of all included patients were reviewed to collect information regarding their ILD (year of diagnosis, most recent pulmonary function test preceding the acute episode, usual oxygen dependency, comorbidities) as well as clinical, radiological and blood sample results at admission.

The severity of the respiratory state was described using the ratio of pulse oximetry and the fraction of inspired oxygen (SpO_2_/FiO_2_) at maximum oxygen supplementation during the hospital stay [13].

The etiological diagnosis of acute dyspnea was determined by the physician in charge of the patient and based on the analysis of the patient’s medical file.

### 2.4. Statistical Analysis

Results are expressed as median and interquartile range [IQR] for quantitative variables and *n* (%) for qualitative variables. To compare LUS1 with LUS2, the Wilcoxon rank-sum test was performed. To compare the LUS characteristics among all patients, the Mann–Whitney test was used. *p*-values < 0.05 were considered significant. Statistical analyses were carried out using R-Studio 2015, Integrated Development for R (RStudio, Boston, MA, USA).

### 2.5. Ethics

Patients were informed and gave oral consent.

This study was conducted in accordance with the Commission Nationale de l’Informatique et des Libertés guidelines and was registered by the local ethics committee of the University Hospital of Tours (n°2022_095).

## 3. Results

We included 24 patients. Nineteen patients were hospitalized in the pulmonology ward and five in the ICU. No patients were excluded because they had received antibiotics for more than 48 h before LUS1 or diuretics more than 6 h before LUS1. The baseline characteristics are summarized in Table 1. Eighteen of the patients were male (75%), with a median age of 72 [66–75] years.

Of the twenty-four patients, nine (37.5%) had idiopathic pulmonary fibrosis (IPF), five (21%) had connective tissue disease-related ILD (two cases of rheumatoid arthritis and three cases of myositis), four (17%) had idiopathic non-specific interstitial pneumonia (NSIP), three (12.5%) had hypersensitivity pneumonia, one had desquamative interstitial pneumonia, one had post-acute respiratory distress syndrome (ARDS) fibrosis and one patient had ILD with a mutation of the ABCA3 gene. Twenty-one patients (87.5%) were previous smokers, but none had chronic obstructive pulmonary disease. Before admission, seven patients (29%) had grade 1 dyspnea on the modified Medical Research Council (mMRC) scale, eight (33%) had grade 2 dyspnea, six patients (25%) reported grade 3 dyspnea and only three (13%) had grade 4 dyspnea. All but three of the patients had pulmonary function tests in the year preceding their admission. The median forced vital capacity (FVC) before admission was 65% of the predicted value (IQR, [53–72]), and the diffusion capacity of the lungs for carbon monoxide (DLCO) was 32% (IQR, [28–38]). Almost half of the patients were under antifibrotic treatment (42%), seven were under long-term corticosteroid treatment (29%), eight had immunosuppressive treatment (33%) and nine patients (37.5%) had long-term oxygen supplementation.

At admission, 83% of patients presented with grade 4 dyspnea on the mMRC scale, and the rest reported grade 3 dyspnea. Fifteen patients (65%) had a new cough or increase in cough, and nine (37%) reported sputum. All patients with pulmonary infection reported a new or worsening cough. The median ratio of SpO_2_/FiO_2_ was 197 (IQR, [118.75–300]). Clinical examination at admission revealed bilateral velcro-type crackles in 18 patients (75%), bilateral non-velcro-type crackles in 3 patients (12.5%), unilateral crackles in 1 patient (4%) and wheezing in 1 patient (4%), and 1 patient (4%) had clear breath sounds. The median reported weight change was a loss of 1.5 kg [−5–0]. Weight change in patients with AE-ILD and infection was significantly different, a loss of 4.25 kg [−5.93–0.9] and 0 kg [−0.75–0.5], respectively (*p*-value = 0.03).

The etiology of the increase in dyspnea was AE-ILD for sixteen patients (67%), infectious pneumonia for six patients (25%) and AHF in two cases (8%). Of the six patients with pulmonary infection, four had viral pneumonia (Respiratory Syncytial Virus, Paramyxovirus influenzae and two with influenza), one had pneumocystis pneumonia and one had a *Streptococcus pneumoniae* infection. One of the patients with viral pneumonia also had a *Klebsiella pneumoniae* co-infection. Regarding therapeutic measures, antibiotics were administered in 19 patients (79%), 9 (38%) had diuretic treatment, corticosteroids were introduced or increased in 19 patients (79%), immunosuppressant treatment was implemented in 2 cases and 1 patient received curative anticoagulants.

Nine patients (37.5%) died during hospitalization with a median survival of 26.5 days [20.5–31.5], of whom eight had AE-ILD and one had infectious pneumonia.

The LUS characteristics of our patients are described in Table 2.

**Figure 1:**
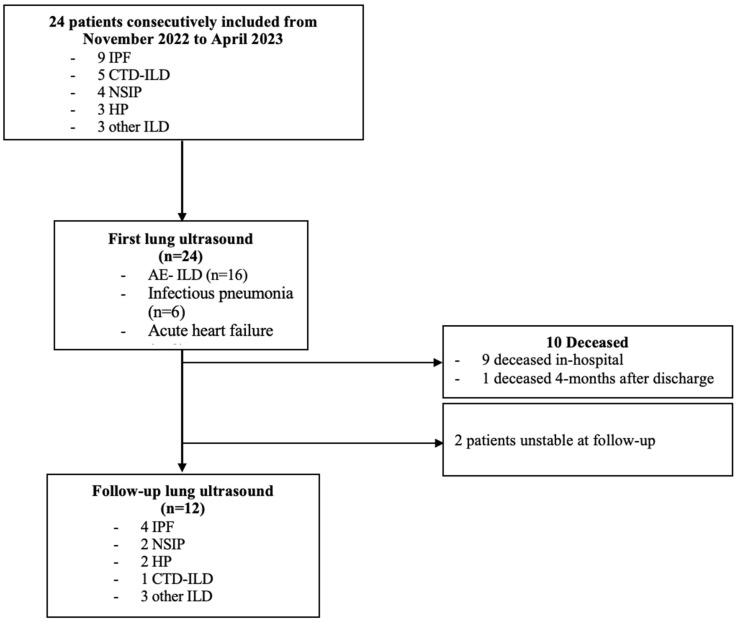
Flow-chart describing the study. E-ILD: acute exacerbation of interstitial lung disease. CTD-ILD: connective tissue disease-related interstitial lung disease. HP: hypersensitivity pneumonia. ILD: interstitial lung disease. IPF: idiopathic pulmonary fibrosis. NSIP: non-specific interstitial pneumonia.

LUS1 was performed in the 24 h following admission in 19 cases. Five patients had LUS1 in the 72 h following their admission. Only 12 out of the 24 patients were eligible for LUS2 as 9 patients had died during hospitalization, 1 had died after hospitalization and before the follow-up consultation, and 2 patients had a deterioration of their respiratory state at the follow-up consultation (Figure 1).

The time between the two LUSs ranged from 38 days to 178 days with a median of 75 days [55;93]. Lung sliding was present in all thoracic areas of all patients in both LUS1 and LUS2. Thickening and/or irregularity of the pleural line were observed in all patients, in most thoracic areas. Twenty-one patients (87.5%) had pleural abnormalities in all thoracic areas on LUS1 versus 100% on LUS2.

**Figure 2:**
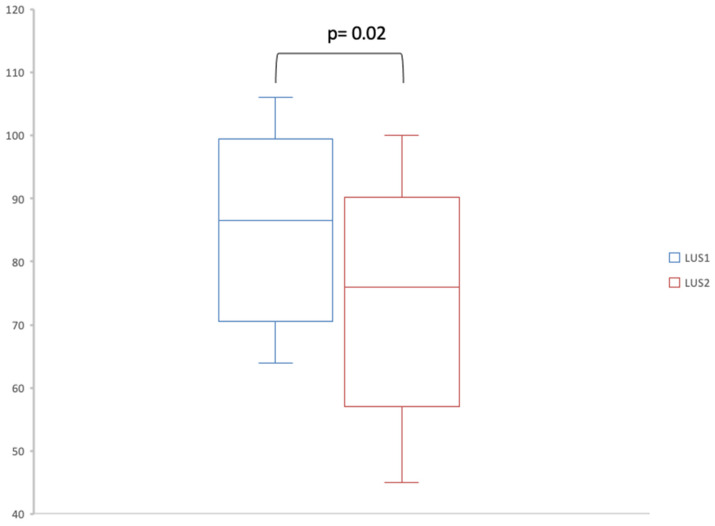
Box plot comparing number of B-lines in LUS1 and LUS2. LUS1: first lung ultrasound. LUS2: lung ultrasound at follow-up.

We compared the number and distribution of B-lines between LUS1 and LUS2 (*n* = 12). There were significantly fewer B-lines on LUS2 (76; IQR, [59–86.75]) than on LUS1 (86.5; IQR, [71.5–94.5]) (*p*-value = 0.02) (Figure 2).

**Figure 3:**
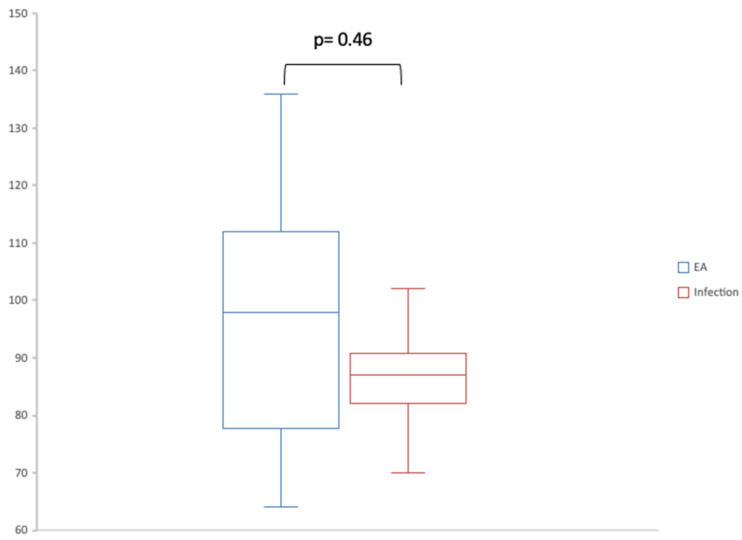
Box plot comparing number of B-lines in LUS1 of patients with AE and those with infection. AE: acute exacerbation. LUS1: first lung ultrasound.

In order to determine the ultrasonographical characteristics of patients with AE-ILD and those with pulmonary infection, we describe the findings of LUS1 for these two groups in Table 3. As shown in Figure 3, there was no significant difference in the number of B-lines between these two groups, with a *p*-value of 0.46 (98 [81.25–110] for AE-ILD and 87 [86.25–87] for infection).

Of the 16 patients with AE-ILD, 3 had A-lines in at least one thoracic area. Four out of the six patients with pulmonary infection had A-lines. LUS1 found pleural effusion in three patients with AE-ILD (18.75%) and no patients with pulmonary infection. It should be noted that the exact causes for the pleural effusions were not determined due to the small amount of pleural fluid not allowing thoracentesis. Two patients with AE-ILD (12.5%) had thoracic areas without pleural line irregularities compared to only one patient with pulmonary infection (17%).

There was no significant difference in the number of B-lines in patients who died (96 [83.75–117.25]) compared to in the survivors (86.5 [71.25–99.5]) (*p*-value = 0.13) (Figure 3).

## 4. Discussion

To the best of our knowledge, this is the first study to describe the LUS of patients with ILD hospitalized with increased dyspnea.

Previous studies focusing on AE-ILD reported a population similar to the patients in our study, with IPF being the most frequent etiology of ILD [14]. Similarly to our cohort, other studies showed that the main cause for hospitalization in patients with ILD is AE-ILD followed by lower respiratory infection and cardio-vascular causes [15]. The mortality in our patients with AE-ILD was very high and consistent with findings observed in other studies [15].

One of the primary findings of this study is that using LUS in these situations is quite feasible with trained physicians. All patients with previously known ILD or the discovery of ILD hospitalized in the Tours University Hospital for increased dyspnea were included in this study. LUS1 was performed in the 24 h following admission in the majority of patients. On the other hand, four of the patients in our study population did not have CT scans at admission or during their hospital stay.

The second main finding of this study is that patients with ILD and acute dyspnea had significantly fewer B-lines after treatment of the acute episode compared to at admission.

B-lines are described as an artifact caused by the partial deaeration of the lung parenchyma [12] (Supplementary Materials). Many studies have shown the correlation between the sum of B-lines present on LUS and the Warrick score combining various interstitial anomalies in chest CT such as ground-glass opacities, septal/subpleural lines, honeycombing, pleural line irregularities and subpleural cysts [16]. There is a scarcity of studies evaluating interstitial syndrome after AE-ILD due to very poor outcomes resulting in death or lung transplantation. In this study, the significant decrease in the number of B-lines following an acute respiratory episode could be an ultrasonographical translation of the decrease in interstitial syndrome.

In addition to ILD, B-lines may be indicative of interstitial syndrome due to infectious pneumonia. In our cohort, three out of the six patients with pulmonary infection (50%) had a viral infection.

One of the secondary objectives of our study was to identify the LUS1 patterns of patients with ILD hospitalized for AE-ILD compared to infectious pneumonia. Clinically, in our cohort, weight loss was significantly more important in patients with AE-ILD compared to in those with pulmonary infection. A 2023 study by Kreuter et al. regarding patients with pulmonary progressive fibrosis showed an association of weight loss and lower BMI with worse outcomes such as an increased risk of acute exacerbation [17]. The comparison of the clinical features of the patients in our cohort shows a forced vital capacity of 56% [43–65] for patients with AE-ILD and 73.5% [69.5–89.5] (*p*-value = 0.07) for patients with infectious pneumonia. These numbers are compatible with those found in various studies involving patients with ILD, highlighting the more severe respiratory state of patients with AE-ILD [18]. Furthermore, there appear to be fewer A-lines in AE-ILD than in pulmonary infections. A-lines disappear when interstitial syndrome and especially B-lines appear [15].

Our patients who died during follow-up had a tendency to have more B-lines than those who survived. There are no data on the prognosis of AE-ILD using LUS, but this result is consistent with data from a meta-analysis by Song et al. in patients with severe viral infection, showing that a high LUS score is associated with increased mortality [19].

Our study has several limitations. First, this is a monocentric study with only 24 patients, of whom only 12 were able to have a follow-up LUS. However, our study was prospective with a follow-up for our patients in the 3 to 6 months after inclusion. Second, there was heterogeneity among the included patients with ILD. This heterogeneity is intrinsic to ILDs, which range from more common etiologies such as IPF to rarer ones like ABCA3 mutation.

Lastly, our study was insufficiently powered to show a difference in LUS features between the different groups.

In conclusion, this study shows a significant decrease in B-lines in survivors in their improved state after a hospitalization for acute dyspnea. This confirms the relevance of LUS in the evaluation of these patients. Our data have not shown a pattern of LUS depending on the etiology of dyspnea.

This pilot study suggests that LUS could be used in the acute state of patients with PID. Further studies are needed to better integrate LUS into the management of these patients.

## Figures and Tables

**Table 1:** Baseline characteristics of patients included in the study.

	All Patients (*n* = 24)	Patients with AE-ILD (*n* = 16)	Patients with Pulmonary Infection (*n* = 6)	*p*-Value
**Baseline characteristics at admission**				
Age, years	72 [66–75]	71.4 [65.8–74.5]	72.3 [67.8–74.7]	0.80
Male gender	18 (75%)	12 (75%)	5 (83%)	1
Type of ILD				
- IPF	9 (37.5%)	5 (31.3%)	3 (50%)	
- Connective tissue-related ILD	5 (21%)	4 (25%)	1 (16.7%)	
- Idiopathic NSIP	4 (17%)	2 (12.5%)	1 (16.7%)	
- Hypersensitivity pneumonia	3 (12.5%)	3 (18.8%)	0	
- Desquamative interstitial pneumonia - Post-ARDS fibrosis	1 (4%) 1 (4%)	0 1 (6.25%)	1 (16.7%) 0	
- ABCA3 mutation	1 (4%)	1 (6.25%)	0	
**Comorbidities at admission**				
Active or previous smokers	22 (91.8%)	15 (93.7%)	5 (83.3%)	0.48
Smoking, pack-years	20 [6.5–30]	17 [6.5–20.5]	28.5 [21.8–31.5]	0.16
BMI	25 [23–29]	25,7 [23.4–28.5]	25.8 [23.6–28.4]	0.91
Immunosuppression	10 (41%)	7 (44%)	2 (33%)	1
Corticosteroid treatment at admission	7 (29%)	5 (31%)	1 (16.7%)	0.63
Immunosuppressive treatment	8 (33%)	4 (25%)	3 (50%)	0.62
Antifibrotic treatment	10 (42%)	10 (62.5%)	4 (66.7%)	0.35
Most recent pulmonary functional test				
FVC % predicted	65 [53–72]	56 [43–65]	73.5 [69.5–89.5]	0.07
DLCO % predicted	32 [28–38]	29 [22–40]	30 [30–37]	0.42
Long-term supplemental oxygen	9 (38%)	8 (50%)	1 (16.7%)	0.33
Usual dyspnea (MRC score)				
1	7 (29%)	6 (38)	1 (17%)	
2	8 (33%)	4 (25%)	3 (50%)	
3	6 (25%)	3 (19%)	2 (33%)	
4	3 (13%)	3 (19%)	0	
**Symptoms at admission**				
Dyspnea (MRC score)				
3	4 (17%)	3 (18.75%)	1 (16.7%)	
4	20 (83%)	13 (81.25%)	5 (83.3%)	
Cough	15 (65%)	9 (56%)	6 (100%)	0.12
Sputum	9 (37%)	7 (38%)	3 (50%)	0.66
Temperature at admission (°C)	36.4 [36.2–37.3]	36.4 [36.2–37.5]	36.95 [36.5–38.1]	0.17
Recent weight change in kg	−1.5 [−5–0]	−4.25 [−5.93–0.88]	0 [−0.75–0.45]	0.03
Maximum oxygen				
supplementation during hospital stay (SpO_2_/FiO_2_)	197 [118.75–300]	134 [117–243]	271 [207–305]	0.24
**Treatments administered**				
Antibiotics	19 (79%)	12 (75%)	6 (100%)	0.54
Diuretic treatment	9 (38%)	7 (44%)	0	0.12
Corticosteroids	19 (79%)	16 (100%)	3 (50%)	0.01
Immunosuppressant therapy	2 (8%)	1 (7%)	1 (16.7%)	1
Anticoagulant treatment	1 (4%)	1 (6.25%)	0	1
**Clinical outcomes**				
In-hospital mortality	9 (38%)	8 (50%)	1 (16.7%)	0.33

AE-ILD: acute exacerbation of interstitial lung disease. ARDS: acute respiratory distress syndrome. NSIP: non-specific interstitial pneumonitis. ILD: interstitial lung disease. IPF: idiopathic pulmonary fibrosis. FVC: forced vital capacity. DLCO: diffusing capacity of the lungs for carbon monoxide. BMI: Body Mass Index. MRC: Medical Research Council. Data is expressed as numbers of patients (%) or medians (interquartile range).

**Table 2:** LUS characteristics of all patients at admission and at follow-up.

	LUS 1 (*n* = 24)	LUS 2 (*n* = 12)
Lung sliding	24 (100%)	12 (100%)
Pleural irregularities in all thoracic areas	21 (87.5%)	12 (100%)
Pleural effusion	4 (17%)	0
Consolidation	2 (8%)	0
Sum of A-lines	0 [0–1]	1 [0–2.25]
Sum of B-lines	86.5 [71.5–94.5]	76 [59–86.75]

LUS: lung ultrasound. Data is expressed as numbers of patients (%) or medians (interquartile range).

**Table 3:** LUS1 characteristics of all patients depending on the etiology of their acute dyspnea.

	All Patients (*n* = 24)	Patients with AE-ILD (*n* = 16)	Patients with Pulmonary Infection (*n* = 6)	Patients with AHF (*n* = 2)
Lung sliding	24 (100%)	16 (100%)	6 (100%)	2 (100%)
Pleural irregularities in all thoracic areas	21 (87.5%)	14 (87.5)	5 (83%)	2 (100%)
Pleural effusion	4 (17%)	0	3 (18.75%)	1 (50%)
Consolidation	2 (8%)	1 (6.25%)	1 (17%)	0
Sum of A-lines	0 [0–1]	0 [0–0]	1 [0.25–1.75]	0.5 [0.25–0.75]
Sum of B-lines	87 [81.25–105]	98 [81.25–110]	87 [86.25–87]	79 [75–82]

AE-ILD: acute exacerbation of interstitial lung disease. Data is expressed as numbers of patients (%) or medians (interquartile range).

## Data Availability

This study was reported to the CNIL advisor to the Tours University Hospital who approved its compliance with CNIL guidelines. The study is registered in the Tours University Hospital’s database with a reference number (n°2022_095).

## Supplementary Materials

The following supporting information can be downloaded at https://www.mdpi.com/article/10.3390/jcm14124159/s1, Video S1: Video of lung ultrasound showing B-lines in a patient with interstitial lung disease.

## Abbreviations

The following abbreviations are used in this manuscript:

AE-ILD	Acute exacerbation of interstitial lung disease
AHF	Acute heart failure
BLUE protocol	Bedside Lung Ultrasound in Emergency protocol
BMI	Body mass index
CT	Computed tomography
CTD	Connective tissue disorder
CXR	Chest X-ray
DLCO	Diffusion capacity of the lungs for carbon monoxide
FiO_2_	Fraction of inspired oxygen
FVC	Forced vital capacity
ICU	Intensive care unit
ILD	Interstitial lung disease
LUS	Lung ultrasound
PE	Pulmonary embolism
SpO_2_	Pulse oxygen saturation
